# Proteomic differences between extracellular vesicles and extracellular vesicle-depleted excretory/secretory products of barber’s pole worm

**DOI:** 10.1186/s13071-023-06092-6

**Published:** 2024-01-12

**Authors:** Fei Wu, Xueqiu Chen, Zhendong Du, Yanqiong Chen, Danni Tong, Jingju Zhang, Yi Yang, Guangxu Ma, Aifang Du

**Affiliations:** College of Animal Sciences, Zhejiang Provincial Key Laboratory of Preventive Veterinary Medicine, Institute of Preventive Veterinary Medicine, Zhejiang University, Hangzhou, 310058 China

**Keywords:** *Haemonchuscontortus*, Excretory/secretory products, Extracellular vesicles, Immune modulation

## Abstract

**Background:**

Components of excretory/secretory products (ESPs) of helminths have been proposed as vaccine targets and shown to play a role in modulating host immune responses for decades. Such research interest is further increased by the discovery of extracellular vesicles (EVs) in the ESPs of parasitic worms. Although efforts have been made to reveal the cargos of EVs, little is known about the proteomic differences between EVs and canonical ESPs released by parasitic worms from animals.

**Methods:**

The total ESPs of *Haemonchus* *contortus* (barber’s pole worm) were obtained by short-term in vitro culturing of young adult worms, and small EVs were isolated from ESPs using an ultracentrifugation method. Data-dependent acquisition (DDA) label-free Nano-LC–MS/MS was used to quantify the proteomic difference between small EVs and EV-depleted ESPs of *H.* *contortus*. Functional annotation and enrichment of the differential proteins were performed regarding cellular components, molecular functions, pathways, and/or biological processes.

**Results:**

A total of 1697 proteins were identified in small EVs and EV-depleted ESPs of *H.* *contortus* adult worms, with 706 unique proteins detected in the former and 597 unique proteins in the latter. It was revealed that proteins in small EVs are dominantly cytoplasmic, whereas proteins in EV-depleted ESPs are mainly extracellular; canonical ESPs such as proteases and small GTPases were abundantly detected in small EVs, and SCP/TAP-, DUF-, and GLOBIN domain-containing proteins were mainly found in EV-depleted ESPs. Compared with well-characterised proteins in small EVs, about 50% of the proteins detected in EV-depleted ESPs were poorly characterised.

**Conclusions:**

There are remarkable differences between small EVs and EV-depleted ESPs of *H.* *contortus* in terms of protein composition. Immune modulatory effects caused by nematode ESPs are possibly contributed mainly by the proteins in small EVs.

**Graphical Abstract:**

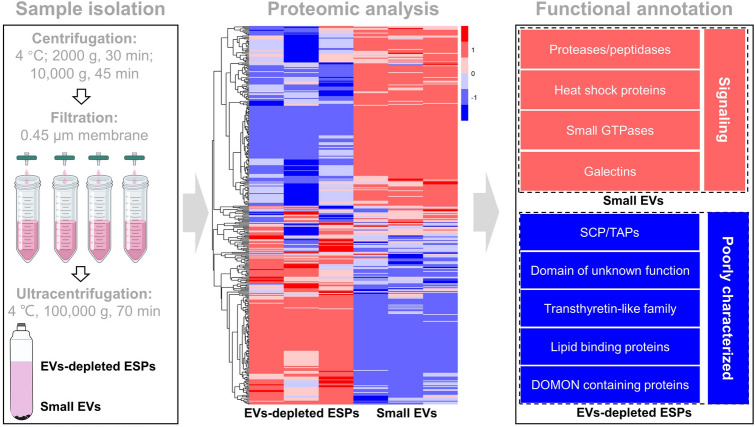

**Supplementary Information:**

The online version contains supplementary material available at 10.1186/s13071-023-06092-6.

## Background

Parasitic worms are considered master manipulators of immune responses within host animals, using a variety of strategies to facilitate long-term parasitism and survival [1]. One of the most successful strategies of parasitic worms to maintain relationships with their hosts is to release excretory/secretory products (ESPs) from the pharynx, intestine, and/or gonad [2]. Indeed, extensive studies have shown that ESPs released by parasitic nematodes, tapeworms or trematodes are involved in a variety of biological processes in host-parasite scenarios [3, 4], such as immunomodulation [1, 5], anticoagulation and antioxidation [6]. Experiments have shown that *Ancylostoma* *ceylanicum*, *Haemonchus* *contortus* and *Dirofilaria* *immitis*-derived ESPs could obviously prolong the prothrombin time (PT) and the activated partial thromboplastin time (APTT), essentially the inhibition of coagulation cascade [7–9]. Particularly, it has been shown that immunisation with ESP-derived proteins or the complex mixture (total ESPs) produced by helminths can induce somewhat protective effects in animals from worm infection [10–13]. For instance, Barbervax, the vaccine for intervention against haemonchosis in lambs, was developed using crude extractions of gut natural proteins (the dominant effectors are H11 and H-gal-GP, which have also been found in ESPs) of *H.* *contortus* [14, 15]. However, this vaccine cannot provide long-term protection for sheep [16–18] and has been proposed to be combined with surface antigen of the infective larvae of *H.* *contortus* [18] for a satisfactory intervention against diseases caused by this important parasite.

In the past decades, ESPs and their functions have been extensively investigated for parasitic worms of major importance [4]. Typically, ESPs are a mixture of multiple substances, including proteins, carbohydrates, lipids and nucleotides. The characterised proteins include but are not limited to proteinases (e.g. metallo-, amino-, serine, cysteine and aspartic proteinases), proteinase inhibitors (TIL, Kunitz, serpin, Kazal type), lectins (C-type lectins and galectins), venom allergen-like proteins (VALs; sperm-coating proteins/Tpx/Sc7, SCP/TAPs), heat shock proteins (HSPs) and transthyretin-like family (TLF, TTR) proteins [2–4, 19]. Some proteins, lipids and particularly nucleotides are usually encapsulated by various extracellular vesicles (EVs; small/middle/large EVs and other vesicles) [20, 21]. Growing evidence has shown that small EVs (i.e. exosomes) are important mechanisms in intercellular, parasite-parasite and host-parasite (interspecies) communication, playing a role in parasitism and manipulation of their host [22, 23]. For example, protozoan species could evade the human anti-infection responses by transferring non-hereditary virulence through EVs and host erythrocytes that could be remodelled after fusing with trypanosome EVs and causing anaemia [24, 25]. However, although the composition of nematode ESPs and associated EVs have been investigated [3, 19, 26, 27], little is known about the differences between EVs and the canonical ESPs, and if any would be derived from independent experiments, hindering an efficient integration.

Here, the proteomic difference between small EVs and EV-depleted ESPs of *H.* *contortus* was unveiled by integrating a short-term in vitro culturing method and a LC-MS/MS analysis of the two compartments. This simultaneous work elucidating the protein cargos of total ESPs and small EVs should underpin the study of host-parasite interaction, particularly the mechanisms underlining host immune modulation by this and related parasitic worms.

## Materials and methods

### Animals and nematodes

Four-month-old helminth-free Hu sheep (*n* = 3) were used to collect the adult worms of *H.* *contortus* as described previously [9, 20]. Briefly, each sheep was injected with dexamethasone sodium phosphate at a dose of 10 mg/sheep intramuscularly daily for a week. Three days after the initial injection (on the 4th day), each sheep was orally infected with about 8000 infective third-stage larvae (iL3s) of *H.* *contortus*. Young adult worms were harvested from the abomasa at 14 days post infection.

### Culture of worms

The collected young adults were washed five times (5 min shaking at each time) with sterilized physiological saline, axenisation fluid (70 mM NaCl, 2.5 mM KCl, 10 mM Na_2_HPO_4_, 2.5 mM NaH_2_PO_4_, 5 mM glucose, 10 U/ml penicillin, 100 U/ml streptomycin, 10 μg/ml amphotericin B, 80 μg/ml ciprofloxacin, 100 μg/ml ampicillin, 40 μg/ml gentamicin; modified from previous studies [28, 29]) and DMEM (containing 1% penicillin-streptomycin-gentamicin), step by step, to minimise contamination. Young adult worms were cultured in vitro using a well-established method [20, 30]. In brief, extensively washed worms were transferred into a T75 cell flask containing 50 ml DMEM and 1% penicillin-streptomycin-gentamicin (1000 worms per 50 ml) and then incubated in a 38 ℃ incubator with 10% CO_2_ for 24 h. Culturing medium was collected for ESP collection.

### Small EVs and EV-depleted ESP isolation

The small EVs and EV-depleted ESPs from *H.* *contortus* were obtained using a well-established protocol [19, 31–34]. First, eggs, dead cells and debris in the ESP collection were removed by centrifugation at 2000*g* for 30 min at 4 ℃. The supernatant was subjected to centrifugation at 10,000*g* for 45 min at 4 ℃ (to separate the middle/large EVs) and then filtered through a 0.45-μm filter and ultracentrifuged at 100,000*g* for 70 min at 4 ℃. The sediment (small EVs) was resuspended in 10 ml pre-cold PBS (Sangon Biotech Co., Ltd., Shanghai, China), centrifuged at 100,000*g* for 70 min at 4 ℃ and then resuspended in 400 μl pre-cooled PBS and stored at − 80 ℃ until use. Small EVs were added with 2% sodium deoxycholate lysate (containing 1 × protease inhibitor; Roche, Basel, Switzerland) and then mixed, heated at 95 ℃ for 10 min and broken using an ultrasonic breaker for 10 min (working for 2 s with 2 s interval). Proteins (the supernatant) from small EVs were collected through centrifugation at 20,000×*g* at 4 ℃ for 15 min. The supernatants were concentrated using a Ultracel-10 regenerated cellulose membrane (Millipore Corp, Bedford, MA, USA) to reduce the volume to 500 μl, then washed with 5 ml pre-cooled PBS twice and further concentrated to 250 μl (EV-depleted ESPs) and stored at – 80 ℃; 8 M urea/50 mM Tris–HCL containing 1 × protease inhibitor was used to generate lysates of EV-depleted ESPs. Then, dithiothreitol (DTT; coolaber, Beijing, China; a final concentration of 10 mM) was individually added into protein samples of small EVs and EV-depleted ESPs, and the tubes containing the mixture were placed in 37 ℃ water bath for 1 h, followed by adding the appropriate amount of iodoacetamide (IAA; Sangon Biotech; a final concentration of 20 mM) and placing the mixture away from light for 30 min.

The concentration of proteins in small EVs or EV-depleted ESPs was measured using a Bradford method according to the manufacturer’s instructions (Beyotime Biotechnology, Shanghai, China). From each sample, 10 μg of protein was boiled, mixed with loading buffer and centrifuged, and then separated on 12% SDS-PAGE gel (Fude Biological Technology, Hangzhou, China) by subjecting it to 120 V constant pressure electrophoresis for 40 min. The gel was stained, decolorised and imaged.

### HPLC fractionation

Protein digestion and reversed-phase HPLC were performed according to the standardised methods previously described [19, 31, 35]. Briefly, 3 μg trypsin was added to 150 μg of each protein, respectively, for digestion by incubating at 37 ℃ for 16 h. The enzymatically digested peptides were then desalted and dried, redissolved in pure water and stored at − 20 °C.

Equal amounts of peptides from each sample were mixed and then diluted with solvent A (5% ACN, pH 9.8) and injected into the column. The peptide mixture was fractionated using a 3.5-μm 4.6 × 150 mm Agilent ZORBAX 300 Extend-C18 column on a Thermo Scientific UltiMate™ 3000 Binary Rapid Separation System (Agilent Technologies, Inc., Santa Clara, CA, USA). The gradient elution was performed at a flow rate of 0.3 ml/min: 5–21% solvent B (97% ACN, pH 9.8) in 38 min, 21.5% to 40% solvent B in 20 min, 40–90% solvent B in 2 min, 90% solvent B for 3 min and 5% solvent B equilibrated for 10 min. The elution peaks were monitored at 214 nm and fractions were collected every minute. The fractions were combined according to chromatograms of the elution peaks. Ten fractions were obtained and then freeze-dried.

### Nano-LC–MS/MS

Proteins in small EVs and EV-depleted ESPs were identified using a Nano-LC–MS/MS method, in accordance with consolidated protocols [32, 33, 36]. In brief, the dried peptide samples were redissolved with 0.1% FA followed by 10 min centrifugation (20,000*g*). The supernatant was collected and injected into a self-loading C18 column (100-μm I.D., 1.8-μm column media particle size, approximately 35-cm column length). Separation was performed by Thermo Scientific EASY-nLC™ 1200 system (Thermo Fisher Scientific Inc., Waltham, MA, USA) at a flow rate of 300 nl/min through the following effective gradient: From 0 to 103 min, 4% solvent B (98% ACN, 0.1% FA) was linearly increased to 27%; 103–111 min, solvent B was increased from 27 to 40%; 111–113 min, solvent B was increased from 40 to 90%; 113–120 min, 90% solvent B. The separated peptides were ionized by a nano-ElectroSpray Ionization (ESI) and then transferred to Orbitrap Exploris™ 480 mass spectrometer (Thermo Fisher Scientific, San Jose, CA, USA) for data-dependent acquisition (DDA) mode detection. Parameter settings were 2.2 kV ion source voltage and 350–1500 *m*/*z* scan range of primary mass spectrometry; resolution was 60,000; normalized AGC Target was 300%, and maximum ion injection time (MIT) was 20 ms. The secondary mass spectrometry fragmentation mode was HCD. The fragmentation energy was set at 32%; resolution was set at 15,000; dynamic exclusion time was 60 s. The starting m/z of secondary mass spectrometry was fixed to 110; the parent ion screening condition for secondary fragmentation was charged 2+ to 6+. Normalized AGC Target was set at standard, and the maximum ion injection time (MIT) was 22 ms.

### Protein identification and quantification

The DAA label-free MS/MS data (raw data) were processed and analysed using MaxQuant (version 2.1.4.0) software [37]. Parameter settings included type: standard; enzyme: trypsin/P; maximum missed cleavages: 2; fixed modification: carbamidomethyl (C); variable modifications: oxidation (M) and acetyl (protein N-term); precursor mass tolerance: 20 ppm; fragment mass tolerance: 0.05 Da; match between runs and second peptide search was enabled. All other parameters were in default. The MS/MS data were searched against protein sequences downloaded from the WormBase ParaSite database (version WBPS18; BioProject PRJEB506). The false discovery rate (FDR) threshold was set as 1% at both PSM (peptide spectrum match) and protein levels. Peptides from either contaminant or reverse were removed.

Protein intensity data were normalized by "medium" method. Hierarchical clustering was performed using pheatmap package (v.1.0.12). T-test was used for statistical differential analysis, with a cut of *P* ≤ 0.05 and fold change ≥ 2 used to determine proteins differentially identified in EVs and EV-depleted ESPs. All analyses were performed in R (v.4.3.1) environment.

### Functional annotation

Prediction of subcellular localisation was performed using the WoLF PSORT [38]. Functional annotations including UniProt [39], eggNOG [40], Gene Ontology [41], KEGG Pathway [42], WikiPathways [43] and Reactome Pathways [44] as well as hypergeometric-based enrichment analysis were performed in R (version 4.3.1) environment. Enrichment analysis and picture drawing of volcano plot, bubble chart and Sankey diagram were performed using a ggplot2 (version 3.4.2) R package.

## Results

### Totals of 1697 proteins are detected in small EVs and EV-depleted ESPs of *H. contortus*

**Fig. 1:**
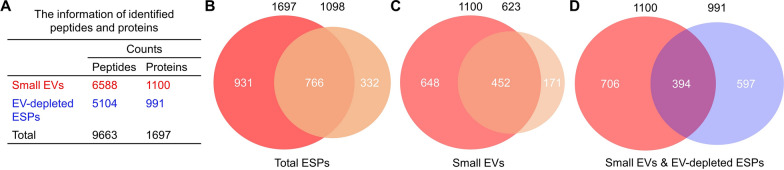
An expansion of proteins identified in the excretory/secretory products (ESPs) of *Haemonchus* *contortus*. **A** The number of peptides and proteins detected in small extracellular vesicles (EVs; indicated in red) and EV-depleted ESPs of *H.* *contortus* (indicated in blue). **B, C** Venn diagrams showing expanded protein cargos in ESPs and small EVs in this work (red) compared previous reports (orange) [19, 32]. **D** A Venn diagram indicating the number of proteins present in small EVs (red) or/and EV-depleted ESPs (blue)

A total of 9663 peptides representing 1697 proteins were identified in the small EVs (*n* = 1100) and EV-depleted ESPs (*n* = 991) released by *H.* *contortus* adult worms in vitro (Fig. 1A; Additional file 1: Table S1). Compared with previous secretome datasets of *H.* *contortus* [19, 32], a total of 931 proteins were newly identified in this work, most of which (*n* = 648) were detected in the small EVs (Fig. 1B, C). By searching major open access databases such as UniProt, eggNOG, Gene Ontology and KEGG Pathway, all 1697 proteins were annotated or described (Additional file 1: Table S1; Additional file 2: Fig. S1).

### There is a remarkable proteomic difference between small EVs and EV-depleted ESPs

There were 706 unique proteins identified in small EVs and 597 unique proteins identified in EV-depleted ESPs of *H.* *contortus* adult worms (Fig. 1D). Specifically, the functional annotation of proteins in small EVs and EV-depleted ESPs is different. These proteins are characterised either comprehensively or briefly by the major open access databases (typically the more annotation the less mystery), and proteins uniquely derived from *H.* *contortus* small EVs and EV-depleted ESPs are probably described by relatively more (≥ 5) and fewer (≤ 4) databases, respectively (Additional file 2: Fig. S1). For instance, proteases (especially cysteine and serine proteases; [45]), heat shock proteins (HSPs) and small GTPases (also known as Ras superfamily; [46]), which are well annotated, are dominantly detected in small EVs (Table 1). By contrast, many more functionally unknown proteins, including domains of unknown function (DUF) and SCP/TAPs/CRISP, and poorly characterised proteins (i.e. DOMON domain-containing, GLOBIN domain-containing and lipid-binding proteins) were found in EV-depleted ESPs of *H.* *contortus* (Table 1).

**Table 1:** Comparison of important proteins identified in small EVs and EV-depleted ESPs

Protein family	Number of proteins		
	Small EVs	EV-depleted ESPs	Common
Protease	**71**	**47**	**57**
Cysteine protease	27	4	16
Aminopeptidase	2	2	11
Serine protease	7	1	3
Metallopeptidase	6	11	4
Aspartyl protease	8	6	11
Other protease	21	23	12
Protease inhibitor	**4**	**10**	**7**
Serine protease inhibitor	3	9	4
Cysteine protease inhibitor	1	1	2
Aspartyl protease inhibitor	0	0	1
Excretory/secretory protein	**12**	**75**	**16**
CRISP/SCP/CAP family	9	59	9
Secreted protein	3	16	7
Protein of unknown function	**19**	**57**	**23**
Lectin	**3**	**6**	**8**
C-type Lectin	0	2	0
Galectin	3	3	6
Other lectin	0	1	2
Lipid-binding protein	**3**	**8**	**13**
Nematode fatty acid retinoid-binding protein	0	2	3
Fatty-acid-binding protein	0	2	3
Saposin	1	2	4
Annexin	1	0	1
START domain-containing protein	0	2	1
Glycolipid transfer protein	1	0	0
Vitellogenin domain-containing protein	0	0	1
Calcium ion-binding protein	**6**	**9**	**17**
EF-hand domain protein	1	4	6
Other calcium ion-binding protein	5	5	11
Heat shock protein	**4**	**2**	**10**
Small GTPase family	**18**	**4**	**3**
Transthyretin-like family	**6**	**8**	**17**
von Willebrand factor	**1**	**6**	**1**
Ig-like superfamily	**2**	**15**	**5**
DOMON domain-containing protein	**1**	**5**	**1**
Globin	**1**	**2**	**8**
Collagen	**7**	**6**	**1**
Fibrinogen	**0**	**4**	**1**

### Differences are also detected in the proteins in both small EVs and EV-depleted ESPs

**Fig. 2:**
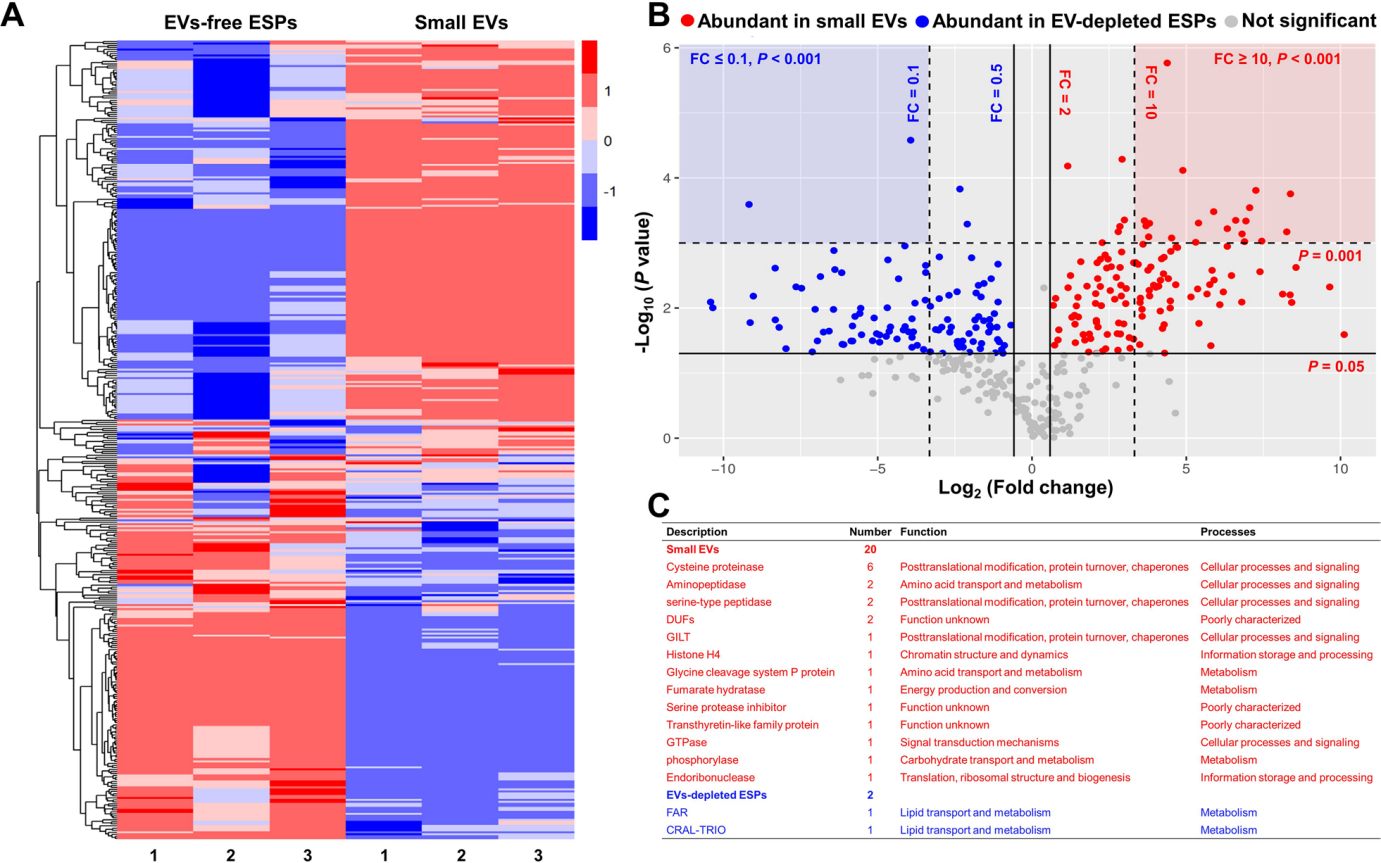
Comparison of proteins identified in both small extracellular vesicles (EVs) and EV-depleted excretory/secretory products (ESPs) from *Haemochus* *contortus*. **A** Heatmap showing the different abundances of proteins detected in small EVs and EV-depleted ESPs of *H.* *contortus*. 1–3: three technical replicates. Red indicates abundant in small EVs or EV-depleted ESPs, and the depth of colour indicates the relative abundance. **B** Volcano plot showing the differential abundance of proteins found in both small EVs (indicated in red) or EV-depleted ESPs of *H.* *contortus* (indicated in blue). **C** Functional description of the most abundant proteins in small EVs (red) or EV-depleted ESPs (blue) based on a criteria of fold change ≥ 10, *P* < 0.001 between the two compartments

**Fig. 3:**
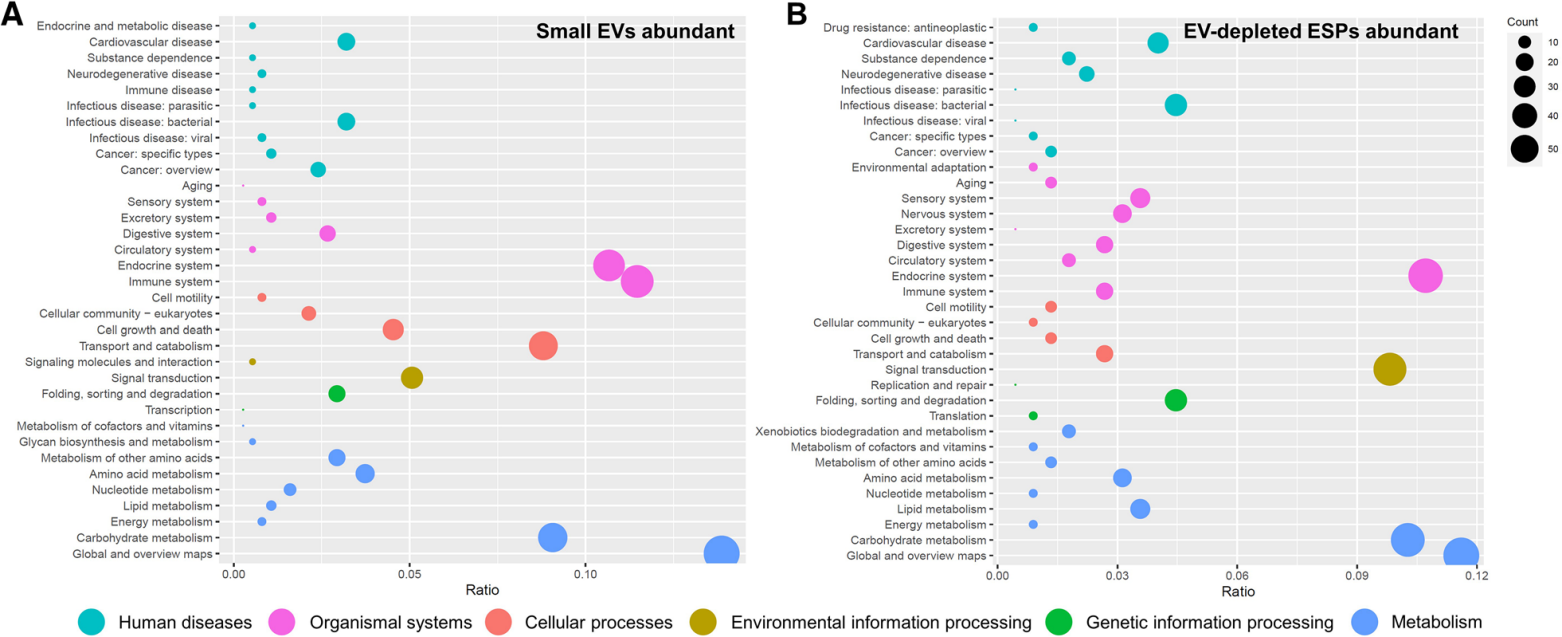
Enriched pathways for proteins abundant in the small extracellular vesicles (EVs) and EV-depleted excretory/secretory products (ESPs) of *Haemonchus* *contortus*. **A** Enriched KEGG pathways of proteins abundant in the small (EVs) of *H.* *contortus*. **B** Enriched KEGG pathways of proteins abundant in the EV-depleted ESPs of *H.* *contortus*. The bubble charts are graphed in R (version 4.3.1) environment using R package ggplot2 (version 3.4.2)

Although 394 proteins were simultaneously identified in small EVs and EV-depleted ESPs of *H.* *contortus* adult worms, there were quantitative differences between these compartments. Most of the proteins showed a dominant abundance in either small EVs or EV-depleted ESPs (Fig. 2A). Differential analysis showed that 129 proteins were significantly dominant in small EVs (log_2_|fold change|≥ 1, *P* < 0.05) and 104 proteins dominant in EV-depleted ESPs (log_2_|fold change|≥ 1, *P* < 0.05) (Fig. 2B). The proteins dominating in small EVs (fold change ≥ 10, *P* < 0.001) were proteinase/peptidase and enzymes, whereas the proteins abundant in EV-depleted ESPs (fold change ≤ 0.1, *P* < 0.001) were lipid-binding proteins [e.g. CRAL-TRIO domain-containing protein and fatty acid and retinol-binding protein (FAR); Fig. 2C]. Proteins related to the host immune system, signal transduction and cellular processes appeared more likely to be abundant in small EVs (Fig. 3).

### Protein cargos of small EVs are the molecules that contribute to host-parasite communication

**Fig. 4:**
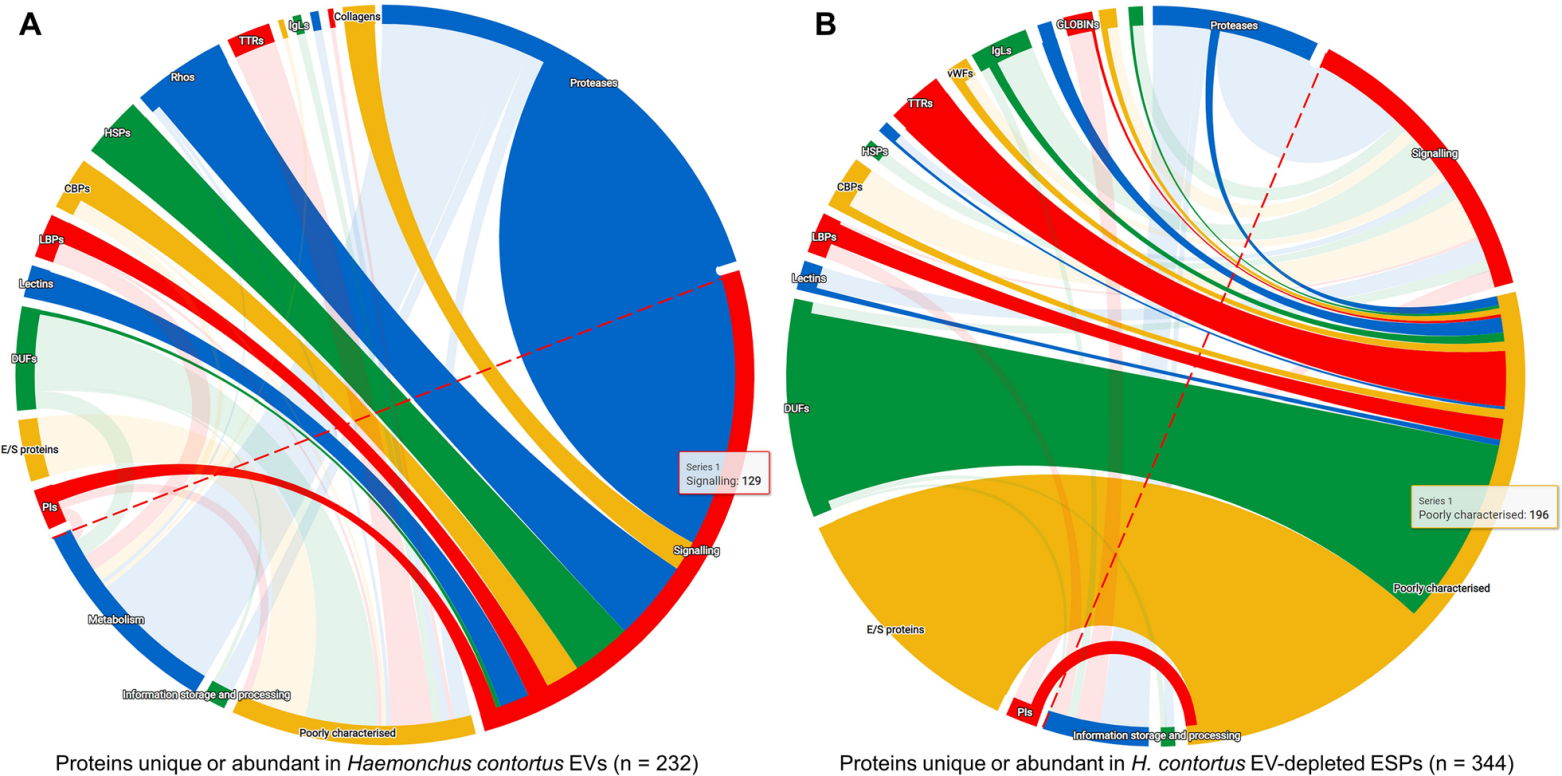
Biological processes for protein families abundant in small extracellular vesicles (EVs) and EV-depleted excretory/secretory products (ESPs) of *Haemonchus* *contortus*. **A** Sankey diagram showing the involved biological processes of protein families unique or abundant in the small (EVs) of *H.* *contortus*. **B** Sankey diagram showing the involved biological processes of protein families unique or abundant in the EV-depleted ESPs of *H.* *contortus*. Protein families are listed above the red dashed line, with biological processes linked. Diagrams are drawn in R environment using a sankeywheel package (version 0.1.0). *PIs* protease inhibitors, *E/S* proteins, excretory/secretory proteins, *DUFs* domain of unknown function, *LBPs* lipid-binding proteins, *CBPs* calcium-binding proteins, *HSPs* heat shock proteins, *Rhos* small GTPases, *TTRs* transthyretin-like family, *IgLs* Ig-like superfamily

More than half of the proteins in small EVs were predicted to be involved in signal transduction and metabolism (Fig. 4A). Moreover, the most abundant proteins in small EVs are cellular processes and signalling or metabolism related (Additional file 1: Table S1; Additional file 3: Table S2). Many of these proteins are proteases/peptidases (Fig. 4A; Table 1), which should play roles in blood-feeding/dwelling helminths, including proteolytic digestion, immune evasion and immunoglobulin degradation properties [45], and in host-parasite interactions. Moreover, HSPs and small GTPases were abundant in this compartment (Table 1), implying a role in nematode infection and parasitism [47, 48], particularly by interfering with intercellular signalling within a host animal (Fig. 4A). In contrast, most EV-depleted ESP origin proteins (including the dominant protein in this compartment) were poorly characterised, if any were TTRs, Ig-like superfamily and FARs (Fig. 4B; Additional file 1: Table S1; Additional file 3: Table S2).

## Discussion

In the past decades, considerable progress has been made in studying parasitic worm ESPs (mainly their protein cargos) and recently extracellular vehicles (including small EVs or exosomes) [1, 2, 19, 49–51]. For example, more than 30 datasets of EVs and their cargos have been described in nematodes, trematodes and cestodes, and the function of EVs and their cargos in host-parasite interactions has been extensively proposed [52, 53]. However, the understanding of functional cargos of EVs (especially exosomes) is still restricted by the reality that the composition of EVs is very complex, and the precise definition of exosomes is to be developed [21]. Therefore, challenges remain in uncovering the mystery of helminth EVs, including without limitation obtaining enough EVs, identifying pan-helminth EV biomarkers and carrying out functional analysis of different EVs (such as exosomes, small EVs and large EVs) and their corresponding cargos [54].

To complement the Minimal Information for Studies of Extracellular Vesicles (MISEV) guidelines, a guidance for helminth EVs research has been proposed [54]. In light of a specific reporting template for helminth EVs (Additional file 4: Table S3), potential issues in the study of *H.* *contortus* EVs have been addressed. For example, 3000 young adults (14 days post infection) were harvested from three artificially infected sheep to address the difficulty of obtaining enough EVs restricted by worm populations; short-term in vitro culturing was conducted to avoid cell/parasite death [55–57]; worms were maintained by DMEM medium with 1% (v/v) penicillin-streptomycin-gentamicin and washed extensively with axenisation fluid and sterilized physiological saline solution to minimise bacterial contamination (Additional file 4: Table S3; [19, 28, 29, 32, 54]). However, we are currently unable to obtain EVs that are released by nematode within a host animal and not clear about the differences between EVs released by nematode in vitro and in vivo. A breakthrough  in vitro culturing systems is needed before these questions can be answered.

It is still difficult to distinguish proteins in large EVs and small EVs (e.g. exosomes) of parasitic worms because of a lack of biomarkers or standards for EVs [54, 58, 59]. Usually, a high-resolution density gradient fractionation method combined with direct immunoaffinity capture has been used to isolate pure exosomes [21]. In the current study, small EVs were separated from large EV-depleted ESPs (large EVs were removed by a 10,000*g* centrifuge) using an ultracentrifugation method. Although no solid evidence supports the existence of universal EV markers for all parasitic worms, some proteins have been proposed as biomarkers for platyhelminth or nematode EVs, respectively. Specifically, M13 metallopeptidase family and actin have been uniquely detected in EVs of nematodes [52, 53]. Members of this protein family were also identified in the small EVs of *H.* *contortus* in the current study. Additionally, 856 proteins were found to be unique or abundant in the small EVs of this parasite, from which potential biomarkers (e.g. TIL type serine protease inhibitor, annexin, glycolipid transfer protein and Ras subfamily small GTPases) might be identified for the small EVs or exosomes of *H.* *contortus*. More evidence could also be found in other parasitic worms [60–65]. Clearly, more experimental studies are warranted to verify the true helminth-, nematode- or even clade-specific markers for EVs.

Nonetheless, proteomic differences were revealed between EVs and EV-depleted ESPs of *H.* *conrotus*. Previously, these proteins have been reported as dominant in quantity or expression abundance in nematode ESPs, including proteases, protease inhibitors, glycoside hydrolases, Transthyretin (TTR)-like protein, lectin (including C-type lectin) and SCP/TAPS proteins, etc. [19, 49–51]. Interestingly, we found that most of these canonical excretory/secretory (E/S) proteins are EVs-sourced/dominant (e.g. proteases/peptidases, protease inhibitors and Ras family members [2, 19, 32]. Parasite ESPs have been predicted to be involved in metabolism and modulation of host immune responses [22, 53, 66–69]. The diversity of cargos in EVs and EV-depleted ESPs indicates distinct roles in regulating the host microenvironment [12, 70]. Particularly, proteases (especially peptidase C1 family and serine-type peptidases), small GTPase superfamily (i.e. Ras family proteins), heat shock protein (HSP) 90 and lipid-binding proteins (spectrin family and annexins) that were linked to vesicular traffic, signalling pathways or other processes inner cells [71, 72] were dominant in small EVs of *H.* *contortus*. In contrast, the SCP/TAP family and conserved secreted proteins (conserved modulators of host responses; [53, 73]), FARs (nematode specific and might play a role in parasitic stages; [74, 75]), von Willebrand factor domain-containing proteins (associated with adhesion, haemostasis and immune responses; [76]), HSP70, Ig-like superfamily proteins (involved in recognition, signalling and immune system; [77]) and many other uncharacterized proteins (i.e. DUFs) were mainly released in a soluble milieu (EV-depleted ESPs) of this parasitic nematodes.

Apart from these exciting findings, there are also some limitations to this work. (i) Due to a lack of biomarkers for nematode EVs, it is still difficult to distinguish proteomic features of small EVs and exosomes released by *H.* *contoruts*. (ii) The reason (technically or biologically) for the common proteins between EVs and EV-depleted ESPs is still not clear. (iii) The uniqueness of proteins in either EVs or EV-depleted ESPs has not yet been verified with experimental approaches (e.g. Western blot and immunoaffinity capture).

## Conclusions

There is a remarkable difference in protein composition between small EVs and EV-depleted ESPs of adult *H.* *contortus*, and most well-characterised “ES proteins/antigens” are dominant in small EVs. These results provide important information showing that the immune modulatory effect of nematode ESPs is likely contributed mainly by the protein cargos of small EVs.

### Supplementary Information

**Additional file 1: Table S1.** Proteins identified in the small extracellular vesicles (EVs) and EV-depleted excretory/secretory products (ESPs) of *Haemonchus* *contortus*.

**Additional file 2: Fig. S1.** An overview of protein annotation for molecules detected in the small extracellular vesicles (EVs; indicated in red) and EV-depleted excretory/secretory products (ESPs) of *Haemonchus* *contortus*. (**A**) A pie chart showing the percentage of proteins functionally annotated or described in databases. (**B**) Annotation of proteins in *Hc*-ESPs in seven major open access databases. These databases are WoLF PSORT [38], UniProt [39], EggNog [40], Gene Ontology (GO; [41]), KEGG Pathway (KEGG; [42]), WikiPathways [43] and Reactome Pathways [44]. GO, including GO_CC for cellular component, GO_BP for biological process and GO_MF for molecular function; Pathway, including KEGG pathway (KEGG_P), Reactome pathways (Reactome_P) and Wikipathways (Wiki_P); EggNog, including EggNog description (EggNog_D), EggNog category (EggNog_C) and EggNog term (EggNog_T); WoLF, subcellularlocation predicted in WoLF PSORT (WoLF_SL); Uniprot, Uniprot description (Uniprot_D). Dots in the corresponding rows indicate annotation, and red and blue connected dots represent characterisation by seven or fewer than four databases, respectively. (**C**) The number of proteins characterised by one or more databases. Number above/on the column means the protein quantity of the corresponding compartment.

**Additional file 3: Table S2.** Selected proteins abundant in the small extracellular vesicles (EVs; top 13) or/and EV-depleted excretory/secretory products (ESPs; top 22) of *Haemonchus* *contortus*.

**Additional file 4: Table S3.** The methodology used to isolate small extracellular vesicles (including exosomes) from the excretory/secretory products of *Haemonchus* *contortus*.

## Data Availability

All data are included within the manuscript and the corresponding Figures and Tables.

## Abbreviations

EVs: Extracellular vesicles
ESPs: Excretory/secretory products
DDA: Data dependent acquisition
FDR: False discovery rate
MIT: Maximum ion injection time
HSPs: Heat shock proteins
TTR/TLF: Transthyretin-like family
TIL: Trypsin inhibitor like
FAR: Fatty acid and retinol binding protein
