# Supplementary figures and images for: Proteomic differences between extracellular vesicles and extracellular vesicle-depleted excretory/secretory products of barber’s pole worm
Source: Parasit Vectors. 2024 Jan 12;17:17. doi: 10.1186/s13071-023-06092-6 (PMC10785392; doi:10.1186/s13071-023-06092-6)

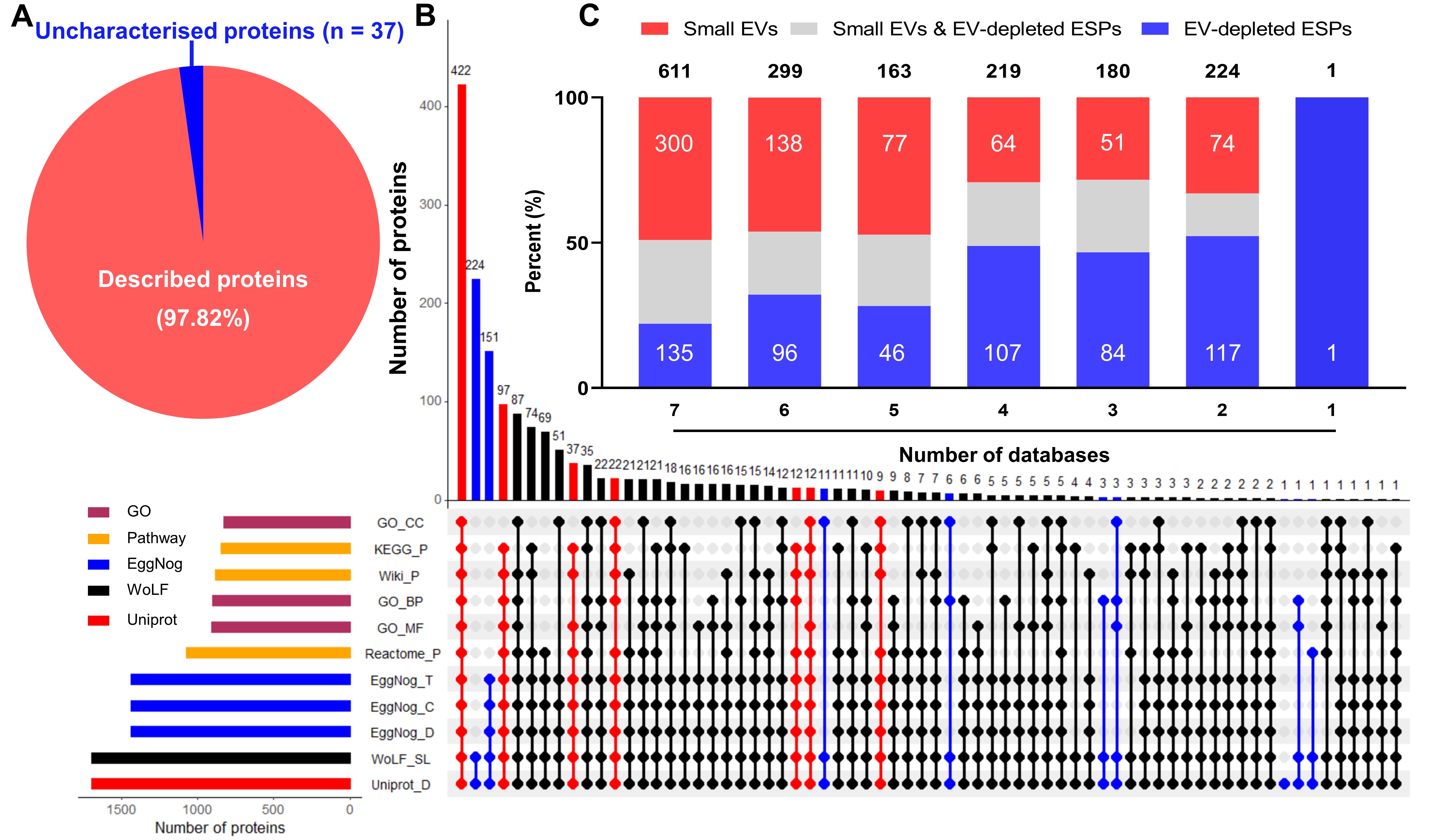

Supplement: Supplementary file 2 — Additional file 2: Fig. S1. An overview of protein annotation for molecules detected in the small extracellular vesicles (EVs; indicated in red) and EV-depleted excretory/secretory products (ESPs) of Haemonchus contortus. (A) A pie chart showing the percentage of proteins functionally annotated or described in databases. (B) Annotation of proteins in Hc-ESPs in seven major open access databases. These databases are WoLF PSORT [38], UniProt [39], EggNog [40], Gene Ontology (GO; [41]), KEGG Pathway (KEGG; [42]), WikiPathways [43] and Reactome Pathways [44]. GO, including GO_CC for cellular component, GO_BP for biological process and GO_MF for molecular function; Pathway, including KEGG pathway (KEGG_P), Reactome pathways (Reactome_P) and Wikipathways (Wiki_P); EggNog, including EggNog description (EggNog_D), EggNog category (EggNog_C) and EggNog term (EggNog_T); WoLF, subcellularlocation predicted in WoLF PSORT (WoLF_SL); Uniprot, Uniprot description (Uniprot_D). Dots in the corresponding rows indicate annotation, and red and blue connected dots represent characterisation by seven or fewer than four databases, respectively. (C) The number of proteins characterised by one or more databases. Number above/on the column means the protein quantity of the corresponding compartment. [file 13071_2023_6092_MOESM2_ESM.jpg]
