# Supplementary material for: Proteomic differences between extracellular vesicles and extracellular vesicle-depleted excretory/secretory products of barber’s pole worm
Source: Parasit Vectors. 2024 Jan 12;17:17. doi: 10.1186/s13071-023-06092-6 (PMC10785392; doi:10.1186/s13071-023-06092-6)
Supplement: Supplementary file 3 — Additional file 3: Table S2. Selected proteins abundant in the small extracellular vesicles (EVs; top 13) or/and EV-depleted excretory/secretory products (ESPs; top 22) of Haemonchus contortus. [file 13071_2023_6092_MOESM3_ESM.docx]

**Additional file 3: Table S2.** Selected proteins abundant in the small extracellular vesicles (EVs; top 13) or/and EV-depleted excretory/secretory products (ESPs; top 22) of *Haemonchus contortus*.

| **Protein names** | **Uniprot_ID** | **Uniprot_description** |
| --- | --- | --- |
| **Small extracellular vescicles (small EVs)** | | |
| HCON_00060770-00001 | A0A7I4Y7Z7 | Saposin B-type domain-containing protein |
| HCON_00047370-00001 | E0YDN3 | Saposin-like protein 1 |
| HCON_00093210-00001 | A0A7I4Y7Z7 | Saposin B-type domain-containing protein |
| HCON_00047720-00001 | A0A7I4Y5P2 | Putative zinc metallopeptidase |
| HCON_00047830-00001 | A0A7I4Y3U5 | Putative zinc metallopeptidase |
| HCON_00152610-00001 | A0A7I4YP38 | Putative zinc metallopeptidase |
| HCON_00093255-00001 | A0A7I5E9W2 | Intestinal prolyl carboxypeptidase 1 |
| HCON_00126500-00001 | A0A7I4YFP2 | Intestinal prolyl carboxypeptidase 2 |
| HCON_00005480-00001 | A0A7I4XSL5 | Cysteine proteinase |
| HCON_00058150-00001 | U6PW36 | Phosphopyruvate hydratase |
| HCON_00154600-00002 | A0A7I4YU60 | Transthyretin-like family protein |
| **EV-depleted ESPs** | | |
| HCON_00055920-00001 | A0A7I5E7W5 | DUF148 domain-containing protein |
| HCON_00128000-00001 | A0A7I4YQ02 | DUF148 domain-containing protein |
| HCON_00060760-00001 | A0A7I4Y6U0 | Saposin B-type domain-containing protein |
| HCON_00060760-00001 | A0A7I4Y6U0 | Saposin B-type domain-containing protein |
| HCON_00079500-00001 | A0A7I4YC10 | 2-iminobutanoate/2-iminopropanoate deaminase |
| HCON_00079500-00001 | A0A7I4YC10 | 2-iminobutanoate/2-iminopropanoate deaminase |
| HCON_00134300-00001 | A0A7I4YQQ1 | GLOBIN domain-containing protein |
| HCON_00134310-00001 | A0A7I5EC57 | GLOBIN domain-containing protein |
| HCON_00134380-00001 | A0A7I4YU12 | GLOBIN domain-containing protein |
| HCON_00134390-00001 | A0A7I4YSC7 | GLOBIN domain-containing protein |
| HCON_00092780-00001 | A0A7I4YF74 | Fatty acid and retinol binding protein 3 |
| HCON_00092800-00001 | A0A7I5E9V4 | Fatty-acid and retinol-binding protein 1 |
| HCON_00092810-00001 | A0A7I4YHU8 | Fatty acid and retinol binding protein |
| HCON_00137520-00001 | A0A7I4YU60 | Transthyretin-like family protein |
| HCON_00137540-00001 | A0A7I4YSM8 | Transthyretin-like family protein |
| HCON_00105200-00001 | A0A7I4YI94 | Glutamate dehydrogenase |
| HCON_00012090-00001 | A0A7I4XWA7 | Acyl-CoA-binding protein domain containing protein |
| HCON_00128780-00001 | A0A7I5EBU5 | Putative cytochrome c |
| HCON_00153430-00001 | A0A6F7Q4K0 | SCP domain-containing protein |
| HCON_00092410-00001 | A0A7I4YHE1 | DH domain-containing protein |
| **Common** |  |  |
| HCON_00137520-00001 | A0A7I4YU60 | Transthyretin-like family protein |
| HCON_00152610-00001 | A0A7I4YZI4 | Cystatin domain-containing protein |

Note: Proteins highlighted in red indicated more abundant than others.
